# Applications of Functional Nanomaterials in Biomedical Science

**DOI:** 10.3390/nano16080461

**Published:** 2026-04-14

**Authors:** Nebojša Đ. Pantelić, Goran N. Kaluđerović

**Affiliations:** 1 Department of Chemistry and Biochemistry, Faculty of Agriculture, University of Belgrade, Nemanjina 6, 11080 Belgrade, Serbia; 2 Department of Engineering and Natural Sciences, University of Applied Sciences Merseburg, Eberhard-Leibnitz-Str. 2, 06217 Merseburg, Germany

## 1. Introduction

Over the past few decades, nanomaterials have emerged as one of the most dynamic areas of modern materials science, attracting significant attention due to their unique physicochemical properties that differ substantially from those of their bulk counterparts. Owing to their nanoscale dimensions, large surface-to-volume ratio, and tunable structural and functional characteristics, nanomaterials offer remarkable opportunities in the development of innovative technologies in chemistry, biology, and medicine. These distinctive features enable improved reactivity, enhanced interaction with biological systems, and the possibility of designing multifunctional platforms for a wide range of applications.

In particular, the integration of nanomaterials into biomedical research has opened new avenues for addressing complex challenges in diagnostics, therapeutics, and disease monitoring. Nanostructured materials have been increasingly explored for targeted drug delivery, controlled release systems, biosensing, imaging and regenerative medicine. Their ability to improve the bioavailability and selectivity of therapeutic agents while minimizing side effects has positioned nanotechnology as a promising strategy for the development of next-generation medical solutions.

At the same time, advances in synthetic strategies and characterization techniques have enabled the rational design of nanomaterials with tailored properties, further expanding their potential applications. Consequently, interdisciplinary research combining chemistry, materials science, biology, and medicine will continue to drive the development of novel nanostructures with improved functionality and performance.

This Special Issue aims to highlight recent progress in the synthesis, characterization, and application of nanomaterials, particularly focusing on their roles in chemical and biomedical research. The contributions gathered in this Issue provide insights into emerging strategies and technological advances that demonstrate the growing importance of nanomaterials in addressing current scientific and medical challenges.

## 2. An Overview of Published Articles

The articles included in this Special Issue illustrate the diversity of current research directions in the field of nanomaterials and demonstrate their broad applicability in biomedical and chemical sciences. The published contributions cover various aspects ranging from the design and characterization of novel nanostructured systems to their potential use in sensing, imaging and therapeutic applications. Together, these studies highlight how advances in nanotechnology continue to provide innovative solutions for improving existing methodologies and developing new strategies for medical and analytical purposes. The contributions include both original research articles and review papers addressing different aspects of nanomaterial design and application. In the following section, a brief overview of the articles published in this Special Issue is presented.

The design and surface functionalization of nanoparticles remain crucial aspects for improving their stability and performance in biomedical applications. In this context, Vilar-Hernández et al. [1] investigated the coating of gold nanoparticles with different phospholipids using a seed growth method. Their results demonstrated that nanoparticle stability strongly depends on the size distribution of both gold nanoparticle seeds and lipid vesicles, while coatings containing phosphatidylglycerol showed promising stability compared with zwitterionic lipids.

Another contribution by Milisavljević et al. [2] focused on the possible application of mesoporous silica nanostructures loaded with triphenyltin(IV) derivatives as potential neuroprotective agents. The study evaluated their inhibitory activity against acetylcholinesterase, which is an enzyme closely associated with the pathophysiology of Alzheimer’s disease. The obtained results indicated that the silica-supported organotin compounds exhibit significant inhibitory activity, highlighting their potential as candidates for the development of novel therapeutic strategies targeting neurodegenerative disorders.

In the field of cancer therapy, Chen et al. [3] reported the development of multifunctional nanodroplets designed to overcome hypoxia-associated resistance in radiotherapy. The authors synthesized oxygen-carrying nanoparticles containing perfluorohexane and superparamagnetic iron oxide, which enhance oxygenation in tumor tissues and promote the generation of reactive oxygen species. These properties contribute to increased DNA damage in cancer cells and improved efficiency of radiation therapy.

Fletes-Vargas et al. [4] explored TiO_2_ nanoparticles loaded with *Polygonum cuspidatum* extract as a potential system for wound-healing applications. The developed nanoparticles exhibited antioxidant and antimicrobial activity, while also demonstrating suitable hemocompatibility and low cytotoxicity. These findings suggest that TiO_2_-based nanocarriers may represent promising platforms for controlled delivery of bioactive compounds in skin regeneration and wound management.

In addition to the original research articles, this Special Issue also includes three review papers that summarize recent developments in important areas of nanomaterials research. A comprehensive overview of calcium phosphate-based coatings functionalized with bioactive compounds for orthopedic applications is presented by Montesissa et al. [5]. Recent advances in coating strategies aimed at improving osseointegration, antimicrobial activity, and long-term implant stability are discussed. Particular emphasis is placed on the incorporation of therapeutic agents, such as growth factors and antibiotics, which can significantly enhance bone regeneration and reduce post-surgical complications.

Emon et al. [6] reviewed recent progress in the development of nanomaterials for bone scaffolds and tissue regeneration, with a focus on their structural and biological characteristics. Various nanostructured systems, including polymeric, ceramic, and composite scaffolds, are examined in terms of their ability to mimic the natural extracellular matrix. Key design parameters such as porosity, mechanical strength, and biocompatibility are highlighted as essential factors for successful applications in regenerative medicine.

An overview of nano-oncologic vaccines as emerging tools for cancer immunotherapy is summarized by Chen et al. [7]. The discussion focuses on nanocarrier systems designed for efficient and targeted delivery of tumor-associated antigens and immunostimulatory agents. These approaches demonstrate significant potential for enhancing immune response activation, overcoming tumor-induced immunosuppression, and improving the overall efficacy of cancer treatment strategies.

## 3. Conclusions

The contributions presented in this Special Issue highlight the continuous progress in the design, characterization, and application of nanomaterials in chemical and biomedical research. The research articles demonstrate how nanostructured systems can be engineered to address diverse challenges, ranging from therapeutic and diagnostic applications to advanced biomedical technologies. The review articles further complement this Special Issue by providing comprehensive insights into current trends and future perspectives, thereby helping to contextualize recent advances and guide ongoing research in the field of functional nanomaterials. Taken together, these works underline the significant potential of nanomaterials to contribute to the development of innovative approaches in medicine and related scientific fields. It is anticipated that the results presented in this Special Issue will encourage further research and foster new directions in the rapidly evolving field of nanotechnology.

## Data Availability

Not applicable.
